# Prenatal Diagnosis in a Fetus With X-Linked Recessive Chondrodysplasia Punctata: Identification and Functional Study of a Novel Missense Mutation in *ARSE*

**DOI:** 10.3389/fgene.2021.722694

**Published:** 2021-09-24

**Authors:** Li Zhang, Haoran Hu, Desheng Liang, Zhuo Li, Lingqian Wu

**Affiliations:** ^1^Center for Medical Genetics, Hunan Key Laboratory of Medical Genetics, Hunan Key Laboratory of Animal Models for Human Diseases, School of Life Sciences, Central South University, Changsha, China; ^2^Hunan Jiahui Genetics Hospital, Changsha, China

**Keywords:** X-linked recessive chondrodysplasia punctata, *ARSE*, prenatal diagnosis, novel mutation, functional experiment, skeletal disease, arylsulfatase E

## Abstract

X-Linked recessive chondrodysplasia punctata (CDPX1) is a rare skeletal dysplasia characterized by stippled epiphyses, brachytelephalangy, and nasomaxillary hypoplasia. CDPX1 is caused by function loss of arylsulfatase E (ARSE, also known as ARSL). Pathogenic mutations in *ARSE* are responsible for CDPX1 in newborns or adults; however, studies have not fully explored prenatal cases. In the current study, a novel missense mutation (c.265A > G) in *ARSE* was identified in a fetus with short limbs using whole-exome sequencing (WES). Bioinformatic analysis showed that the variant was pathogenic, and RT-qPCR, Western blot, and enzymatic assays were performed to further explore pathogenicity of the variant. The findings showed that the variant decreased transcription and protein expression levels and led to loss of enzymatic activity of the protein. The novel mutation c.265A > G in *ARSE* was thus the genetic cause for the phenotype presented by the fetus. The current study presents a prenatal case in Chinese population using functional analysis of *ARSE*, which helps the family to predict recurrence risks for future pregnancies and provides more information for understanding this rare condition. The findings show that WES is a feasible method for prenatal diagnosis of fetuses with CDPX1.

## Introduction

*ARSE* is located on chromosome Xp22.33 which encodes arylsulfatase E (ARSE, MIM *^∗^*300180) (Matos-Miranda et al., 2013). ARSE is a member of the arylsulfatase subfamily (Uchimura et al., 2006), and its precursor is a 60-kD proenzyme. The precursor is subjected to N-glycosylation, to form a mature 68 kD protein and is localized in the Golgi body (Daniele et al., 1998). ARSE plays a role in removing sulfate groups from substrates containing phenolic rings (Cosma et al., 2003). Mutations in *ARSE* affect hydrolysis of sulfate groups of downstream intracellular substrates, resulting in X-linked recessive chondrodysplasia punctata (CDPX1, MIM #302950).

CDPX1 is a rare X chromosomal recessive inherited cartilage calcification disease (Braverman et al., 1993). Overall morbidity of CDPX1 is estimated to be one in every 100,000 people, and studies report that males are the only probands (Deepthi et al., 2018). Detailed studies have not been conducted on CDPX1 owing to the small low incidence. Main clinical symptoms of CDPX1 in affected males include stippled epiphyses, shortening of distal phalanges, and nasomaxillary hypoplasia (Nino et al., 2008; Matos-Miranda et al., 2013). Although skeleton of most patients improves by adulthood, some have severe medical condition including respiratory involvement (Wolpoe et al., 2004), cervical stenosis with instability (Garnier et al., 2007; Vogel and Menezes, 2012), mixed conductive and sensorineural hearing loss (Vrečar et al., 2015), cardiac anomalies, and intellectual disability (Braverman et al., 1993). Currently, there is no radical treatment for CDPX1; therefore, early treatments of manifestations are the only effective management approaches for clinical cases. Treatment approaches such as nasal stents, reconstructive surgery, cervical collar, and spinal fusion are costly and significantly affect the quality of life of the patient. Therefore, diagnosis should be performed in time.

Diagnosis of CDPX1 is based on radiographic findings and a hemizygous *ARSE* pathogenic variant identified by molecular genetic testing (Braverman et al., 1993). Genetic tests are required to make an accurate diagnosis. Molecular testing techniques have been significantly improved in the past. Whole-exome sequencing (WES) is a powerful tool for identification of potential mutations in suspected diseases, mainly for prenatal diagnosis. The first prenatal case with CDPX1 was reported in 2019 (He et al., 2019) by a study that combined ultrasound results and WES without functional experiments on the *ARSE* mutation. The findings showed that WES is an effective tool for prenatal diagnosis and screening of CDPX1. However, not all prenatal case can be fully and accurately diagnosed through WES without conducting functional experiments.

The current study explored a non-consanguineous healthy Chinese couple with a fetus who had short limbs as shown by prenatal ultrasound screening. WES was performed to verify the diagnosis of the fetus. Findings from bioinformatic and functional experiments indicated that a novel missense variant in *ARSE* gene was the key pathogenic factor for the phenotype observed, indicating diagnosis of CDPX1 for the affected fetus. The findings from the current study increase mutation spectrum of *ARSE* and showed that the family has recurrence risks or CDPX1 in future pregnancies.

## Materials and Methods

### Patients and Clinical Materials

A primipara was attended at our hospital at 24^+1^ weeks of gestation for routine ultrasound examination. Ultrasound examination results indicated that her male fetus had short limbs. The first-trimester ultrasound scan showed no obvious abnormalities. However, at 24 weeks of the gestation, the ultrasound testing revealed significant short limbs of the fetus but normal for brain, heart, face, nuchal translucency, nasal bone, clip, spine, and so on. Previous medical histories of the parents were negative. The parents had no history of exposure to alcohol or drugs such as warfarin. The study protocol was approved by the Ethics Committee of Hunan Jiahui Genetic Specialized Hospital. Written informed consent was obtained from all family members participating in the current study.

### Whole-Exome Sequencing

Whole-genome sequencing was performed to confirm the pathogeny of the index fetus. Genomic DNA of the parents and the affected fetus was extracted from blood and amniocyte separately. Exomes were hybridized and captured by the xGen Exome Research Panel of Integrated DNA Technologies (IDT, San Diego, CA, United States) and sequenced on Novaseq6000 platform (Illumina, San Diego, CA, United States) with 150-bp pair-end reads. The sequencing reads were aligned to the human reference genome (hg19/GRCh37). VeritaTrekker^®^ Variants Detection System was used for variant calling. Variant annotation was conducted using ANNOVAR (Wang et al., 2010) and Enliven^®^ Variants Annotation Interpretation System. Candidate pathogenic variants were confirmed by Sanger sequencing.

### *In silico* Analysis

Different bioinformatic tools were used to explore the potential functional effect of the novel missense mutation including: Mutation Taster^1^ (Schwarz et al., 2014), Polyphen-2^2^ (Adzhubei et al., 2010), SIFT,^3^ PROVEAN^4^ (Choi and Chan, 2015), Varcard^5^ (Li et al., 2018), and FATHMM.^6^ Clustal Omega webserver was used to explore conservation of the variant site. A total of 12 species with different degrees of evolution including human were randomly selected, and homologous proteins of each species were aligned. The proportion of amino acid S at the variant site was determined, and the possibility of pathogenicity of the variant was determined based on the conserved value.

### Homology Modeling and Structural Analysis

Crystal structure of ARSE protein has not been determined through experimental methods. Therefore, the online software Swiss Model was used to build a homology model of the wild-type and mutant of ARSE, and the protein structure was analyzed by comparison with similar structures (Waterhouse et al., 2018).

ARSE is highly correlated with ARSC in molecular evolution, thus it may be derived from this gene through repetition (Sardiello et al., 2005). In addition, the ARSC is highly homologous to ARSE. The two proteins are structural proteins, and the ALP_like domain is highly conserved (Ghosh, 2004). Therefore, ARSC, a member of the human sulfate esterase family, was used as the template (PDB Entry: 1P49) for homology modeling.

### Plasmid Construction and Transfection

Coding sequence (CDS) of the *ARSE* gene (NM_000047.3) was cloned in a pCMV vector (pCMV-N-HA-Kan/Neo) and fused with HA tag using T4 DNA ligase method. Mutagenesis for the missense mutation was performed using Mut Express II Fast Mutagenesis Kit V2 (Vazyme). Primers used in mutagenesis are presented in Supplementary Table 1. Sequencing verified the identities of the variant clones, and analysis showed that there was no other mutation present in the vectors.

293T cell line was used for *in vitro* analyses. Before transfection, cells were plated in six-well plates and cultured following standard procedures to 70% confluence. Transfection was performed by lipofection using Lipofectamine 3000 (Invitrogen), and 2 μg of the plasmid was transfected to achieve maximum expression. Cells transfected with the wild-type and mutated *ARSE* vectors represented the normal control group and the experimental group, respectively, cells transfected with empty vector represented the negative control, and untransfected cells were used as blank control. Cells were harvested at 48 h after transfection. All transfections were performed in triplicate. Total RNAs were extracted using TRIzol reagent (Invitrogen, Carlsbad, CA, United States). Total proteins were extracted using RIPA (Beyotime, Jiangsu, China) and Trixon-X100 (Beyotime). Extraction of RNA and protein were performed following the manufacturer’s protocol.

### RT-qPCR

cDNA was synthesized using Revert Aid First Strand cDNA Synthesis Kit (Thermo Fisher Scientific, Waltham, MA, United States) following the manufacturer’s instructions. qPCR was performed using the Maxima SYBR Green/ROX qPCR Master Mix (2×) (Thermo Fisher Scientific) and ASA 9600. Relative gene expression levels were determined using 2 (^−^ΔΔCt) method after normalizing the levels to the level of GAPDH mRNA. Primers used for qPCRs are presented in Supplementary Table 2.

### Western Blot

Western blot was performed using standard protocol. Protein quantification was performed using Pierce^TM^ BCA Protein Assay Kit (Thermo Fisher Scientific). Each sample was prepared to a concentration of 1 μg/μl and boiled for 10 min with loading buffer. Fifteen microliters protein aliquots were run on 10% SDS-PAGE (Beyotime). The proteins were then transferred onto a polyvinylidene difluoride (PVDF) membrane. The membranes were blocked with skimmed milk powder (5%) in 0.1% TBST for 1 h. The membranes were then incubated overnight at 4°C with primary antibody against HA-tagged peptide (1:2,000 dilution; Beyotime) and GAPDH (1:5,000; Cell Signaling, Danvers, MA, United States). In addition, membranes were incubated with horseradish peroxidase-conjugated secondary antibody (1: 10,000 dilution; Cell Signaling). Protein levels were normalized based on GAPDH band density. Immunoblots were probed using ECL Plus kit (Thermo Fisher Scientific). Blot bands were quantified by densitometry using ImageJ software (ImageJ 2).

### ARSE Enzymatic Assay

Enzyme activity of ARSE was determined following a method described previously with slight modification (Chang et al., 1985; Franco et al., 1995; Matos-Miranda et al., 2013). The enzymatic reaction system was as follows: the total system of 100 μl consisted of 0.05 mol/L Tris HCl buffer (pH 7.5), 20 μg Trixon-X100 lysate, and 0.2 mmol/L 4-MUS (Sigma-Aldrich, Saint Louis, MO, M7133, United States). The prepared system was incubated in a 96-well plate at 37°C. The reaction was terminated by addition of 1.8 ml glycine carbonate buffer (pH 10.7). Fluorescence was then measured under 365 nm excitation light and 460 nm emission light for all samples of each group. ddH_2_O and pure substrate without addition of enzyme was measured separately to determine the background fluorescence signal. The background fluorescence signal of the pure substrate group was subtracted during data analysis. Samples were analyzed at 0.5, 1, 1.5, 2, and 2.5 h to determine the production of 4-MU, and the enzymatic reaction process curve of the product yield over time was plotted.

### Statistical Analysis

All data were expressed as the mean ± standard deviation, and statistical comparisons between two groups were evaluated by Student’s *t*-test using Prism software (GraphPad Prism 8.2.1). *p* < 0.05 was considered statistically significant.

## Results

### Identification of a Novel Hemizygous Variant in *ARSE*

**FIGURE 1:**
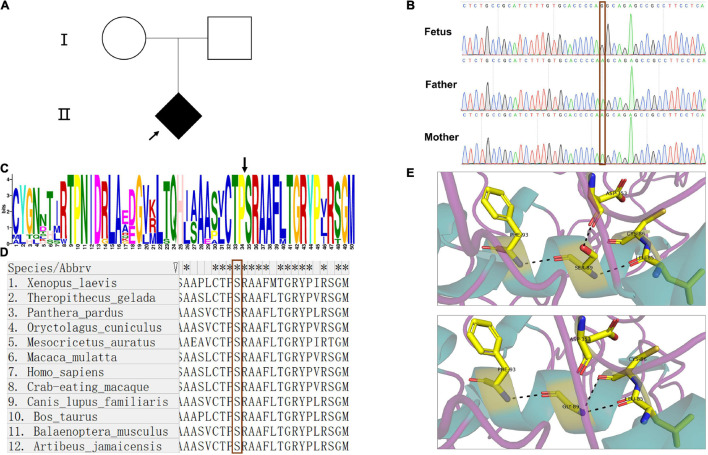
Pedigree of the family, Sanger sequence, and bioinformatics analysis. **(A)** Pedigree chart of the patient family; the arrow indicates the index fetus. **(B)** The results of Sanger sequence showed that the mutation in ARSE is a *de novo* mutation; the red frame indicates the site of the mutation. **(C**,**D)** Motif analysis and alignment analysis of the mutation site show the high conservation of the mutated site, the black arrow in **(C)** and red frame in **(D)** indicates the mutated site of amino acid. **(E)** 3D structural model of wild-type and mutated ARSE proteins, which shows the hydrogen bond change in mutated protein. The protein model is stained with secondary structure, and the flexible structures are magenta, the α-helixes are green, and the β-pleated sheets are orange. The protein residues and key interaction residues affected by the mutation are shown in a stick pattern, and the atomic color of the stick residues is as follows: carbon, yellow; nitrogen, blue; and oxygen, red. Hydrogen bonds between proteins are indicated by a black dotted line. The upper is wild-type ARSE. The lower shows the mutation of p.Ser89Gly.

WES was performed to confirm the pathogeny of the index fetus (Figure 1A). The findings from WES showed that the fetus carried a hemizygous variant c.265A > G (p.Ser89Gly) in *ARSE* gene. The variant in *ARSE* was confirmed by Sanger sequencing, and the mutation in *ARSE* is confirmed by Sanger sequencing, which is absent in parents, revealing that it is a *de novo* mutation (Figure 1B). The variant has not been reported previously in all public databases.

Detailed information on the variant obtained through *in silico* analysis is presented in Table 1. Analysis using prediction algorithms showed that the novel variant deleterious. Amino acid sequence alignment and motif analysis indicated high evolutionary conservation of residues was affected by variants in *ARSE* (Figures 1C,D and Table 1).

**TABLE 1:** Overview of the ARSE mutation c.265A > G *in silico*.

**Algorithm**	**Score**	**Prediction**
ExAC	NA	NA
SIFT	0.000	Damaging
Polyphen-2	1.00	Probably_damaging
Mutation taster	1.000	Protein feature affected/splice site changes
PROVEAN	−3.92	Deleterious
FATHMM	−3.65	Damaging
Varcard	0.86	Damaging
Conservation	100% S in 12 species	Damaging

Three-dimensional structural models of wild-type and mutated ARSE proteins built using Swiss Model were visualized in PyMOL (Schrodinger, 2015), and the structural changes of proteins near the missense mutations were observed (Figure 1E). The findings showed that p.Ser89Gly was located at the end of the first α helix region on the sulfatase domain, which is the functional domain. In addition, a new hydrogen bond was formed between glycine at position 89 and cysteine at position 86 in the mutant and a hydrogen bond between glycine at position 89 and aspartic acid at position 353 was lost, which may have led to a change in the spatial structure of the entire peptide chain.

Analysis using the American College of Medical Genetics and Genomics (ACMG) and the Association for Molecular Pathology (AMP) (Richards et al., 2015) shows that this variant is potentially pathogenic (PS2 + PM1 + PM2 + PP3), details can be found in Supplementary Table 3.

### The Mutant Shows Decreased Expression Level at the Transcription and Protein Level

Furthermore, 293T cells were transfected with pCMV-HA-ARSE^WT^ and pCMV-HA-ARSE^MT^, and the relative quantitative analysis of mRNA and protein expression was performed. Cells without any treatment were used as the blank-control group, and cells without pCMV-HA transfection were used as the negative control group.

**FIGURE 2:**
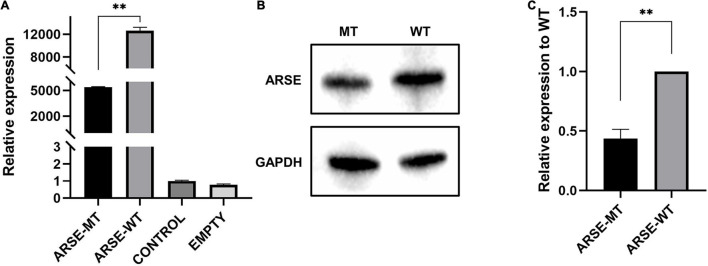
Expression analysis of wild-type and mutated ARSE. **(A)** Transcriptional expression analysis of wild-type and mutated ARSE shows the decrease transcriptional level of this mutation. **(B)** The target band integration diagram demonstrates the same molecular weight between wild-type and mutated ARSE and the decreased expression level of mutated protein. **(C)** The relative expression level of mutant protein obtained after normalization of wild-type protein expression level, which indicates the low expression level of mutated protein. Statistical analysis was performed by GraphPad Prism. ARSE-WT, wild-type ARSE; ARSE-MT, mutated ARSE; EMPTY, empty plasmid vector group; CONTROL, blank-control of 293T cells. The difference between ARSE-WT and ARSE-MT is tested by Student’s *t*-test. ^∗∗^*p* < 0.1.

Transcription level of wild-type and mutated *ARSE* gene was quantitatively analyzed by RT-qPCR with GAPDH as the reference gene. The findings showed that mRNA expression levels were approximately 40% lower in variant carriers compared with the level in the normal control (*p* < 0.01) (Figure 2A).

Analysis of protein expression showed that wild-type and c.265A > G mutant proteins had bands slightly less than 70 kD in size, indicating that the mutant protein can be modified to a mature size of 68 kD similar to the wild-type protein (Figure 2B). However, gray-scale statistical analysis results showed that the relative expression levels of mutant protein were significantly lower compared with the level of the wild-type protein (*p* < 0.01) (Figure 2C).

### Mutant ARSE Has No Enzymatic Activity

**FIGURE 3:**
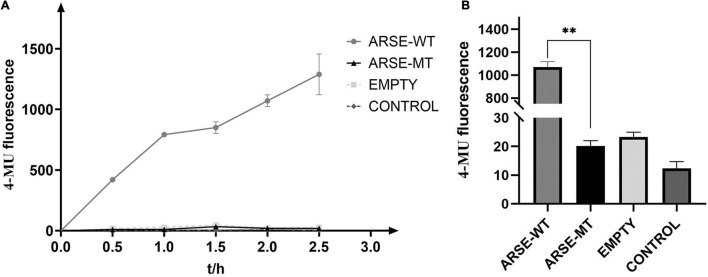
Determination of enzyme reaction of ARSE shows the mutated protein have negligible arylsulfatase E activity. **(A)** ARSE enzymatic reaction process curve. **(B)** Comparison diagram of enzyme activity shown at 2 h after ARSE enzymatic reaction. Statistical analysis and mapping were performed by GraphPad Prism. ARSE-WT, wild-type ARSE; ARSE-MT represents the mutated ARSE, EMPTY represents the empty plasmid vector group, and CONTROL represents the blank-control of 293T cells. The difference between ARSE-WT and ARSE-MT is tested by Student’s *t*-test. ^∗∗^*p* < 0.1.

Enzymatic reaction process curve of the product yield over time was plotted (Figure 3A). The findings showed that the mutant type of ARSE almost coincidences with the negative control and blank-control indicating that it had completely lost its enzymatic activity. On the contrary, the wild type of ARSE effectively desulfurated 4-MUS to generate 4-MU. Data were recorded every 2 h (Figure 3B). Fluorescence signal intensity of 4-MUS for the wild-type ARSE was 1,071 ± 48.6, which was significantly higher compared with the fluorescence intensity of the mutant ARSE (20 ± 1.9). The findings showed no statistical difference between enzymatic activity of the mutant ARSE and the empty vector group (23 ± 1.7). These findings indicate that the wild-type protein expressed by 293T cells has good enzymatic activity, whereas the mutant protein had negligible arylsulfatase E activity.

## Discussion

The current study presents a case of a non-consanguineous healthy Chinese couple with a male fetus. The case presented with short limbs as shown by prenatal ultrasound screening. Ultrasound analysis was not enough to make a clinical diagnosis. Therefore, to explore the genetic etiology for this family, WES was performed on the family members. The findings showed that the fetus carried a *de novo* missense variant of c.265A > G in *ARSE* gene. To the best of our knowledge, this variant has not been reported in public databases. Bioinformation analysis showed that the variant was potentially pathogenic (PS2 + PM1 + PM2 + PP3). RT-qPCR, Western blot, and enzymatic assays showed a decrease in transcription and protein expression level as well as loss of enzyme activity, indicating the pathogenicity of the mutation. These findings implied that the mutation c.265A > G was the potential genetic cause for the phenotype of the fetus.

The first prenatal case with CDPX1 was reported in 2019 (He et al., 2019). The fetus was at 23 weeks of gestation, and ultrasound analysis showed hypoplasia of the midface and the nose, accompanied by spinal stenosis and secondary ossification center was reported in early stages. WES analysis showed presence of a novel missense mutation of c.640G > A in *ARSE* gene. Bioinformatic function prediction analysis showed that the novel missense mutation c.640G > A was pathogenic. However, not all fetuses present with the physical features that can be detected by ultrasound in prenatal period. In the current study, ultrasound analysis only showed the short limb, thus a final diagnosis could not be made based on the ultrasound analysis. Therefore, WES and functional experiments were performed to explore the cause of this phenotype of the fetus. The current study presents the first prenatal diagnosis with functional experiments of CDPX1 and provides a successful example of prenatal diagnosis of CDPX1.

Although 47 *ARSE* mutations have been reported at present, only a few of them have been functionally tested. Several new mutations are present with unknown significance in clinical detection. Therefore, it is imperative to expand the spectrum of mutation with clear pathogenicity. Previous functional studies explored *ARSE* mutation focusing on the enzyme activity but did not explore mRNA and protein expression levels (Matos-Miranda et al., 2013), whereas some only performed RT-qPCR without enzymatic assays (Casarin et al., 2009). The findings of the current study showed both expression level and enzymatic assay are important in making a clinical diagnosis of similar cases. Findings from the current study and previous studies (Casarin et al., 2009) showed that missense mutations can affect transcription levels, which may be a potential pathogenic mechanism. In our research, though the expression of mutated protein drops by nearly half, there are still proteins that remained while the mutated protein only has negligible arylsulfatase E activity. The decrease of enzyme activity was much greater than that of protein expression. Therefore, the protein structure change may be the main cause for the loss of ARSE function. In addition, enzyme activity is an important indicator to explore pathogenic mechanism. Enzymatic assays should be performed for cases with a positive correlation to the severity of phenotype. The use of enzyme activity for clinical diagnosis of CDPX1 is highly recommended once there are enough studies showing enzyme activity-phenotype correlation.

Notably, unlike normal missense mutation, the novel mutation reported in the current study showed changes at the mRNA expression level and loss of protein enzymatic activity.

It was previously believed that point mutations including missense mutations or synonymous mutations do not have significant transcriptional level effects. However, previous studies report that some single nucleotide polymorphism sites can change mRNA expression levels (Kimchi-Sarfaty et al., 2007; Brest et al., 2011). Studies report that change in RNA structure promotes degradation of RNA (Sharma et al., 2019). In the current study, a possible explanation is that the missense mutation of c.265A > G changed the secondary structure of RNA causing instability of the mutant mRNA. The secondary structure of the wild-type and mutated mRNA were predicted to verify this postulation (see Supplementary Figure 1). The findings showed a significant change at sequences near the mutation site. Further studies should be conducted on *in vitro* chemical probing of RNA secondary structure using the selective 2’-hydroxyl acylation analyzed by Primer Extension (SHAPE) protocol.

In addition, splicing or frameshift mutations, but not missense mutations, are reported to significantly affect the structure of proteins, resulting in loss of enzymatic activity. To explore the mechanism behind loss of activity for the mutant protein, analysis was conducted on the protein structure. The findings showed that the mutation had several effects on the protein structure. Notably, a new hydrogen bond was formed between glycine at position 89 and cysteine at position 86 in the mutant protein, which may lead to continuation of the α helix. In addition, transformation of cysteine residues at 86 to 3-oxalanine (also known as C-formylglycine) was observed which is a key catalytic residue for the catalytic activity of sulfatase. Formation of new hydrogen bonds may affect modification of this cysteine residue. Moreover, the hydrogen bond between glycine at position 89 and aspartic acid at position 353 was lost, which may lead to a change in the spatial structure of the whole peptide chain. These changes indicate that the mutation significantly affects the protein structure, resulting in inactivation of the mutant ARSE.

Although prenatal diagnosis of skeletal dysplasia depends on ultrasound, there are several diseases that share similar physical features. To improve accuracy of diagnosis, molecular diagnosis should be performed (Li et al., 2020). Development of novel genetic technologies and advances in molecular genetic tests such as next-generation sequencing (NGS) have significantly improved prenatal diagnosis. The findings of the current study indicate that NGS technologies, such as WES, are feasible and effective for prenatal diagnosis, mainly for fetuses who have limited clinical manifestation to make a definite diagnosis. The current study reports the first prenatal case of CDPX1 in Chinese population using functional experiments, which indicates that the family has recurrence risks of CDPX1 for future pregnancies and provides more information on diagnosis and screening of this rare disorder.

In summary, the current study presents a novel missense mutation of *ARSE* (c.265A > G) for the first time using WES and determined its pathogenicity for the affected family. Genetic analysis and functional studies expand the spectrum of *ARSE* mutations and present an effective prenatal diagnosis of *ARSE* for the affected fetus. The findings of the study indicated that WES is a useful tool for diagnosing of cases with chondrodysplasia punctata detected by prenatal ultrasound and is an effective method for prenatal screening of CDPX1 in early gestation.

## Data Availability Statement

The data presented in the study are deposited in the open database. The Genome Sequence Archive (GSA) under accession number HRA001195 (https://ngdc.cncb.ac.cn/gsa-human/submit/hra/subHRA001746/finishedOverview).

## Ethics Statement

The studies involving human participants were reviewed and approved by the Ethics Committee of Hunan Jiahui Genetic Specialized Hospital. Written informed consent to participate in this study was provided by the participants’ legal guardian/next of kin.

## Author Contributions

LW, DL, and ZL designed the experiments. LZ and HH carried out the experiments. HH analyzed the experimental results. ZL, LZ, and HH wrote the manuscript. All authors listed approved it for publication.

## Conflict of Interest

The authors declare that the research was conducted in the absence of any commercial or financial relationships that could be construed as a potential conflict of interest.

## Publisher’s Note

All claims expressed in this article are solely those of the authors and do not necessarily represent those of their affiliated organizations, or those of the publisher, the editors and the reviewers. Any product that may be evaluated in this article, or claim that may be made by its manufacturer, is not guaranteed or endorsed by the publisher.

## Footnotes

1
http://www.mutationtaster.org/

2
http://genetics.bwh.harvard.edu/pph2/

3
http://sift.jcvi.org/

4
http://provean.jcvi.org/index.php

5
http://varcards.biols.ac.cn/

6
http://fathmm.biocompute.org.uk/
