# Supplementary material for: Prenatal Diagnosis in a Fetus With X-Linked Recessive Chondrodysplasia Punctata: Identification and Functional Study of a Novel Missense Mutation in ARSE
Source: Front Genet. 2021 Sep 24;12:722694. doi: 10.3389/fgene.2021.722694 (PMC8498588; doi:10.3389/fgene.2021.722694)
Supplement: Supplementary file 1 [file Data_Sheet_1.docx]

Supplementary Material

# Supplementary Figure and Tables

## Supplementary Figure

**
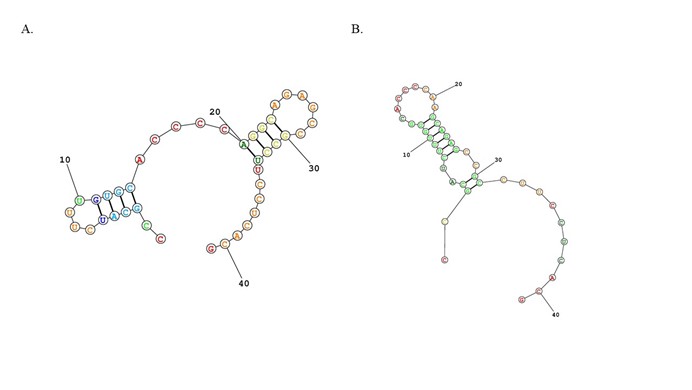
**

**Figure S1.** Prediction of the secondary structure of wild type and mutated mRNA by RNAstructure showed that the structure change highly near the mutation site. A. Wild type ARSE. B. Mutated ARSE.

## Supplementary Tables

**Table S1 *ARSE* CDS amplification primers and mutagenesis primers**

| name | sequence |
| --- | --- |
| *ARSE*-CDS-F | 5’-ccggaattccggATGTTACATCTGCACCATTCTTGTTTGTG-3’ |
| *ARSE*-CDS-R | 5’-gctctagagcTTATTGTGGGTCATCTTCCCTAAGGC-3’ |
| *ARSE*-Mut-F | 5’ATCTTTGTGCACCCCAgGCAGAGCCGCCTTCCTCACGGGCAGATACCCTGTGCGATCAGGGATG-3’ |
| *ARSE*-Mut-R | 5'CcTGGGGTGCACAAAGATGCGGCAGAGATGTGTTGGGTCAGC -3' |

For CDS primers the lowercase base is the protective base and restriction sites; for Mut primers lowercase base means the mutation site

**Table S2 RT-qPCR primers**

| name | sequence | amplified length |
| --- | --- | --- |
| *ARSE*-q1-F | 5’-CTGTACTGCTAAGTTTGGCACC-3’ | 249 |
| *ARSE*-q1-R | 5’-CCTGATCGCACAGGGTATCTG-3’ |  |
| *ARSE*-q2-F | 5’-GATGGTTTCCAGCATTGGTTACC-3’ | 157 |
| *ARSE*-q2-R | 5’-TGGCTGACTCACAGTTGAGAC-3’ |  |
| *ARSE*-q3-F | 5’-ATGCCACTGGACTCATTGGA-3’ | 147 |
| *ARSE*-q3-R | 5’-CAGGTTGACACGCTTCTCTG-3’ |  |
| *GAPDH*-q1-F | 5’-GGAGTCAACGGA TTTGGTCG-3’ | 174 |
| *GAPDH*-q1-R | 5’-TCCTGGAAGATGGTGA TGGG-3’ |  |

**Table S3 Evidence list of American College of Medical Genetics and Genomics (ACMG)**

| Category | Evidence |
| --- | --- |
| PS2 | *De novo* (both maternity and paternity confirmed) in this fetus with the disease and no family history |
| PM1 | Located in a critical and well-established functional domain without benign variation. |
| PM2 | Absent from controls in Exome Sequencing Project, 1000 Genomes Project, or Exome Aggregation Consortium. |
| PP3 | Multiple lines of computational evidence support a deleterious effect on the gene and gene product. ( Clustal Omega: High conservation; Mutation Taster, Polyphen-2, SIFT , PROVEAN, , FATHMM: deleterious) |

The variant is evaluated as “Likely pathogenic” (1 Strong (PS2) and 2 moderate (PM1, PM2)) according to ACMG Standards and Guidelines.
